# The transcription factor AebHLH89 activates *AeGMP1* transcription to regulate L-ascorbic acid accumulation in kiwifruit (*Actinidia eriantha*) revealed by genome-wide association study

**DOI:** 10.1093/hr/uhag116

**Published:** 2026-04-06

**Authors:** Lu Chen, Dongfeng Jia, Yansong Liu, Huan Gao, Guanglian Liao, Jipeng Mao, Zhu Gao, Xiaobiao Xu

**Affiliations:** Kiwifruit Institute/College of Agronomy, Jiangxi Agricultural University, Nanchang, Jiangxi 330045, China; Jiangxi Provincial Key Laboratory of Plantation and High Valued Utilization of Specialty Fruit Tree and Tea/Institute of Resources and Environment, Jiangxi Academy of Sciences, Nanchang, Jiangxi 330096, China; Jiangxi Provincial Institute of Traditional Chinese Medicine, Nanchang, Jiangxi 330046, China; Kiwifruit Institute/College of Agronomy, Jiangxi Agricultural University, Nanchang, Jiangxi 330045, China; Kiwifruit Institute/College of Agronomy, Jiangxi Agricultural University, Nanchang, Jiangxi 330045, China; Kiwifruit Institute/College of Agronomy, Jiangxi Agricultural University, Nanchang, Jiangxi 330045, China; Kiwifruit Institute/College of Agronomy, Jiangxi Agricultural University, Nanchang, Jiangxi 330045, China; Jiangxi Provincial Key Laboratory of Plantation and High Valued Utilization of Specialty Fruit Tree and Tea/Institute of Resources and Environment, Jiangxi Academy of Sciences, Nanchang, Jiangxi 330096, China; Jiangxi Provincial Key Laboratory of Plantation and High Valued Utilization of Specialty Fruit Tree and Tea/Institute of Resources and Environment, Jiangxi Academy of Sciences, Nanchang, Jiangxi 330096, China; Kiwifruit Institute/College of Agronomy, Jiangxi Agricultural University, Nanchang, Jiangxi 330045, China

## Abstract

*Actinidia eriantha*, a kiwifruit species endemic to China, produces fruits with notable nutritional, medicinal, and economic value, particularly due to its high L-ascorbic acid (L-AsA) content. However, the regulatory mechanisms underlying L-AsA accumulation in its fruit remain poorly understood. This study meticulously measured L-AsA levels of fruits in 216 *A. eriantha* accessions from natural populations and performed a genome-wide association study, through which we identified significantly associated lead single nucleotide polymorphisms and insertion deletions, and characterized a key candidate gene *AebHLH89*, *AePPR*, *AePP2Ab*, and *AePHL1* involved in the positive regulation of L-AsA accumulation. Functional experiments showed that overexpression of *AebHLH89* significantly enhanced L-AsA accumulation, while its silencing *via* virus-induced gene silencing markedly decreased L-AsA levels. Yeast one-hybrid assay and dual-luciferase assay preliminary revealed that AebHLH89 could bind to the *AeGMP1* promoter and activates its transcription, thereby upregulating the L-AsA biosynthesis pathway and promoting L-AsA synthesis and accumulation. These findings provide valuable genetic resources for molecular marker-assisted breeding in kiwifruit and contribute to germplasm innovation. Simultaneously, the identification of key regulatory genes enhances our understanding of L-AsA metabolism and lays a theoretical foundation for the genetic improvement of kiwifruit.

## Introduction

China, with its unparalleled diversity of *Actinidia* germplasm resources, is often regarded as the natural genebank for kiwifruit [1]. These species have evolved under the selective pressures of long-term environmental adaptation, resulting in extensive phenotypic and genetic diversity both within and between species. During the domestication and breeding processes, cultivated germplasm types of *Actinidia* with high yield, strong resistance, and superior quality have gradually emerged. *Actinidia eriantha* is a Chinese endemic *Actinidia* species and also a particularly valuable wild resources used in kiwifruit breeding programs [2]. Its fruit possesses extremely high nutritional, medicinal, ornamental, and economic value, and it has the potential to become a significant cultivar following *A. chinensis* and *A. deliciosa*. However, most *A. eriantha* resources grow in wild or semi-wild environments, resulting in diverse fruit qualities. Given this, excellent fruit quality has become a primary goal in kiwifruit breeding, as it not only enhances the market value of *A. eriantha* fruits to meet consumer demands but also drives the high-quality and efficient development of the industry.

Among these, high levels of L-ascorbic acid (L-AsA) content are one of the defining characteristics of *A. eriantha*. L-AsA possesses important functions in living organisms, including nutritional enhancement and antioxidant effects [3–5]. For plants, L-AsA serves not only as a potent nonenzymatic antioxidant that scavenges reactive oxygen species (ROS) and free radicals under biotic and abiotic stresses—particularly during photosynthetic processes—but also participates in cell growth, division, and the biosynthesis of plant hormones [6–9]. L-AsA is also an essential nutrient for human health. However, humans have lost the ability to autonomously synthesize L-AsA due to a mutation in the key enzyme responsible for the final step of its biosynthesis. Consequently, humans must obtain L-AsA from L-AsA-rich fruits and vegetables. Securing sufficient daily L-AsA intake from fresh produce remains a challenge in both developed and developing countries [10–12].

Owing to its exceptional L-AsA content, *A. eriantha* has emerged as a valuable model for investigating the distribution patterns and accumulation mechanisms of L-AsA in plants [13]. In kiwifruit, L-AsA predominantly accumulates in developing fruits and declines progressively during ripening [14]. The maintenance of elevated L-AsA levels is largely governed by biosynthetic pathways—such as the L-galactose, D-galacturonic acid, degradation, and recycling routes—along with associated regeneration processes. Key genes involved in these pathways have been identified, including *GGP* [15], *GalDH* [16], and *GalLDH* [17] in the biosynthetic pathways, as well as *DHAR*, *MDHAR* [14], and *AO* [18] in the recycling pathways. Although the L-AsA biosynthesis pathway in higher plants is well characterized, its regulatory network remains highly complex. The synthesis and metabolism of L-AsA are regulated by multiple factors, including external factors (light exposure [16, 19], ambient temperature [20], and plant hormones, such as ethylene, abscisic acid (ABA), and gibberellin [20–22]), as well as internal genetic determinants. Nevertheless, the molecular basis for the high L-AsA content in *A. eriantha* fruits, including the key regulatory genes and/or transcription factors modulating L-AsA levels, has yet to be fully elucidated.

Genome-wide association study (GWAS) has been successfully applied to the study of quality and key gene mining in horticultural crops, such as the genes affecting fruit flavor and firmness in tomato [23, 24], the genes determining stone cells in pear [25], the Aux/IAA regulator for flesh firmness in watermelon [26], and the identification of candidate genes related to single fruit weight and L-AsA content in *A. eriantha* hybrids [18], and related to fruit firmness and mototerpenoids in grape [27, 28]. In this study, we collected fruits and leaves from 216 *A. eriantha* individuals at physiological maturity from natural population with the same or similar habitats in the Luoxiao Mountains (Ganzhou, Jiangxi Province, China) [29]. Building upon a previously established dataset for comprehensive quality evaluation of this *A. eriantha* population [30], we constructed fruit L-AsA trait datasets for the 216 *A. eriantha* accessions and performed GWAS using high-quality single nucleotide polymorphisms (SNPs) and insertion deletions (InDels) generated *via* whole-genome resequencing. This study aims to identify SNPs/InDels and candidate genes associated with L-AsA. The findings will provide valuable genetic resources for molecular marker-assisted breeding and facilitate germplasm innovation in kiwifruit.

## Results

### Sequencing, quality assessment, and variants of 216 *A. eriantha* accessions

High-quality whole-genome sequencing data were generated from 216 *A. eriantha* accessions, yielding 1.55 Tb of raw sequence data (average 7.33 Gb per individual, Table S1). After quality filtering, we obtained 1.51 Tb of clean data (average 7.14 Gb per individual) with excellent sequence quality (Q20 = 97.88%, Q30 = 93.27%) and normal GC content distribution (average 36.99%, range 35.12–48.30%). The 10× depth sequencing reads showed high alignment efficiency (96.37%) to *A. eriantha* cv. ‘Ganlv 1’ reference genome [31], demonstrating both sequence similarity and uniform coverage. These results show that the quality of the whole-genome resequencing meets the requirements for the subsequent analysis. Genome-wide variant identification and distribution analysis revealed 9 190 904 high-quality variants across the tested *A. eriantha* genome, comprising 1 790 395 SNPs (average density: one SNP every 248 bp) and 7 400 509 InDels (average density: one InDel every 60 bp). Chromosomal distribution analysis showed uniform variant dispersion, with averages of 61 738 SNPs and 255 190 InDels per chromosome (Fig. S1). This comprehensive and abundance variant dataset, with its high-marker density and genome-wide coverage, meets the requirements for subsequent population genetic and association analyses.

### Population structure analysis and linkage disequilibrium decay

**Figure 1:**
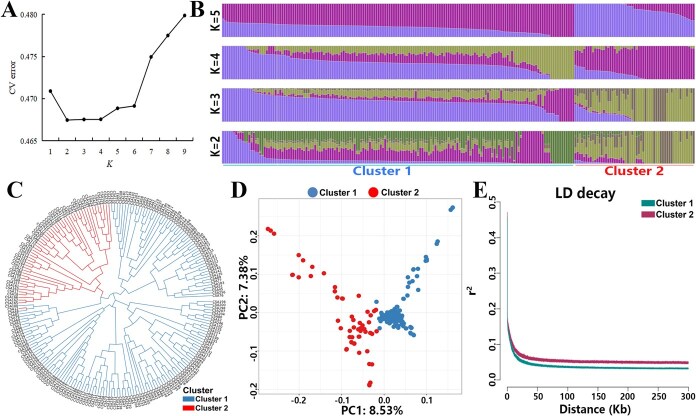
Population structure analysis. (A) Cross-validation error analysis; (B) genetic structure map based on population structure analysis, the horizontal axis shows the different germplasm numbers, the vertical axis shows the probability of germplasm being grouped at a certain *K* value, different colors represent different groups; (C) phylogenetic tree; (D) PCA diagram; (E) model of LD decay.

Our analysis of 1 790 395 high-quality SNPs from 216 *A. eriantha* accessions revealed clear genetic stratification within the population. Initial structure analysis using Admixture identified *K* = 2 as the optimal grouping, dividing the population into two subgroups (161 and 55 accessions), with higher *K* values (3–5) showing increasing admixture that suggested ongoing gene flow (Fig. 1A and B). This division was consistently supported by three independent analyses: phylogenetic tree construction showed distinct clustering with branch lengths reflecting genetic divergence (Fig. 1C); principal component analysis (PCA) clearly separated the two subgroups, with principal components subsequently used for GWAS covariate adjustment (Fig. 1D); and linkage disequilibrium (LD) decay analysis revealed similar patterns in both subgroups, with *r*^2^ values decreasing rapidly within 10 kb before stabilizing (Fig. 1E). The concordance among these different analytical approaches strongly validates the existence of two genetically distinct subgroups in the population, with limited but detectable gene flow between them.

### Descriptive statistics of L-ascorbic acid content among 216 *A. eriantha* accessions

A natural population comprising 216 *A. eriantha* accessions was evaluated for fruit L-AsA content (Table S2). The fruit L-AsA content across the 216 *A. eriantha* accessions ranged from 0.28 to 23.86 mg·g^−1^, with a mean value of 8.28 mg·g^−1^. This trait exhibited a high coefficient of variation (CV) of 55.52% and a Shannon’s genetic diversity index (*H*′) of 5.22 (Table S3), indicating substantial variation and rich genetic diversity within the tested population. The boxplot, normal distribution curve (Fig. S2), and analyses of skewness and kurtosis (Table S3) collectively demonstrated that the L-AsA content followed a normal distribution in this population. These findings confirm that the selected population is suitable for a GWAS.

### The selection of models for genome-wide association study

**Figure 2:**
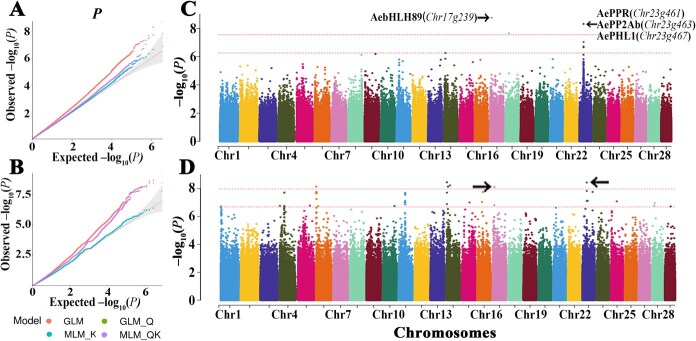
GWAS analyses of L-AsA trait in 216 *A. eriantha* accessions based on SNP and InDel markers. (A) Q–Q plots for four association models based on SNPs; (B) Manhattan plots for MLM-QK model based on SNPs; (C) Q–Q plots for four association models based on InDels; (D) Manhattan plots for MLM-QK model based on InDels.

To assess the impact of different models on association results, we conducted GWAS using four models. We compared the association performance of the models using quantile–quantile (Q–Q) plot (Fig. 2A and B). Because the Bonferroni significance threshold was too conservative, two slightly lower GWAS signal thresholds were chosen for this study [−log_10_(*P*) > 6.27 (0.05 level of significance) and −log_10_(*P*) > 7.54 (0.01 level of significance)]. The Q−Q plots for the MLM-Q and MLM-QK models showed that observed *P*-values aligned with expected values at lower −log_10_(*P*) and diverge only at higher values, indicating minimal false positives and reliable results. To identify models suitable for associating fruit nutritional quality traits in the test population, the association performance of four models (GLM, GLM-Q, MLM-K, and MLM-QK) was compared. The GLM, GLM-Q, MLM-K, and MLM-QK models associated 24, 7, 3, and 7 significant SNPs and 100, 76, 1, and 76 significant InDels, respectively (Figs S3 and S4). The MLM-QK model included population structure and genetic relationship matrices as covariates, accounting for both factors and better controlling false positives in association analysis compared to the GLM model. Considering the correlation results and the advantages of each model, the MLM-QK model was deemed more suitable for the tested population and was used for subsequent analysis.

### Genome-wide association study

The exceptionally high L-AsA content is a defining characteristic of *A. eriantha* fruit. At the 0.05 significance level (−log_10_(*P*) > 6.27), the MLM-QK model identified seven SNPs significantly associated with L-AsA and 28 protein-coding genes were located within the candidate intervals 50 kb upstream and downstream of the seven SNPs (Fig. 2A and C, and Table S4). Meanwhile, 76 InDels were significantly associated with L-AsA and 193 protein-coding genes were located within the candidate intervals (Fig. 2B and D and Table S5). Interestingly, in the results of GWAS, 12 commonly associated potential candidate genes were identified within the candidate intervals of SNP/InDel variation sites significantly associated with chromosomes 17 and 23. Through gene alignment and annotation in public databases and review of previous reports, four candidate genes highly associated with L-AsA were further screened out. Specifically, *Chr17g239* (bHLH transcription factor, *AebHLH89*) [32], *Chr23g461* (pentatricopeptide repeat protein, *AePPR*) [33], *Chr23g463* (serine/threonine-protein phosphatase 2A regulatory subunit B′ gamma-like, *AePP2Ab*) [34], and *Chr23g467* (PHR1-LIKE1-like, *AePHL1*) [35]. Among them, *Chr17g239* (*AebHLH89*) was located 34.02 kb downstream of SNP Chr17:2255503 (−log_10_(*P*) = 8.74, 0.01 level of significance) and 31.30 kb downstream of InDel Chr17:2258226 (−log_10_(*P*) = 8.12, 0.01 level of significance). *Chr23g461*, encoding a pentatricopeptide repeat protein (designated *AePPR*), was positioned 32.57 kb downstream of SNP Chr23:6820142 (−log_10_(*P*) = 8.32, 0.01 level of significance) and 31.30 kb upstream of InDel Chr23:6826303 (−log_10_(*P*) = 8.44, 0.01 level of significance). *Chr23g463*, predicted to encode a serine/threonine-protein phosphatase 2A regulatory subunit B′ gamma-like protein (designated *AePP2Ab*), colocalized with both SNP Chr23:2255503 (−log_10_(*P*) = 8.74, 0.01 level of significance) and InDel Chr23:6826303 (−log_10_(*P*) = 8.44, 0.01 level of significance). *Chr23g467*, encoding a PHR1-LIKE1-like protein (designated *AePHL1*), was situated 13.15 kb downstream of SNP Chr23:6891421 (−log_10_(*P*) = 6.70, 0.05 level of significance) and 49.66 kb downstream of InDel Chr23:6826303 (−log_10_(*P*) = 8.44, 0.01 level of significance).

### Expression of candidate genes in fruit developmental stages

To investigate the association between candidate genes and L-AsA accumulation in *A. eriantha*, we analyzed the expression level of *AebHLH89*, *AePPR*, *AePP2Ab*, and *AePHL1* during fruit development in *A. eriantha* cv. ‘Ganmi 6’. All four genes peaked in expression at 15 days after flowering (DAF), followed by stable levels from late developmental stages to physiological maturity (Fig. S5A and B). Notably, their expression trends paralleled the temporal changes in L-AsA content, and a strong positive correlation was confirmed (*P* < 0.05, Fig. S5C), with the expression pattern of *AebHLH89* showing the highest coherence.

### Functional analysis of candidate genes in kiwifruit and tomato

**Figure 3:**
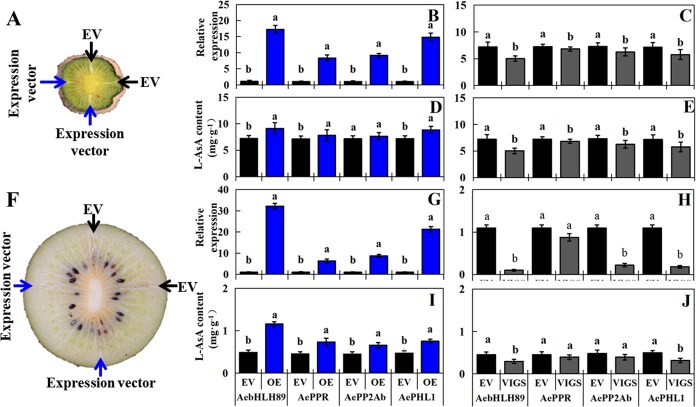
Transient expressing of *AebHLH89*, *AePPR*, *AePP2Ab*, and *AePHL1* in kiwifruit. (A) Transient overexpressing and silencing *AebHLH89*, *AePPR*, *AePP2Ab*, and *AePHL1* in ‘Ganlv 1’ at 105 DAF; relative expression (B and C) and L-AsA content (D and E) of *AebHLH89*, *AePPR*, *AePP2Ab*, and *AePHL1* after transient overexpression and silencing expression in ‘Ganlv 1’, respectively; (F) transient overexpressing and silencing *AebHLH89*, *AePPR*, *AePP2Ab*, and *AePHL1* in ‘Jinyan’ at 120 DAF; relative expression (G and H) and L-AsA content (I and J) of *AebHLH89*, *AePPR*, *AePP2Ab*, and *AePHL1* after transient overexpression and silencing expression in ‘Jinyan’, respectively. Different letters indicated the significant differences (*P* < 0.05).

Transient overexpression of *AebHLH89*, *AePPR*, *AePP2Ab*, and *AePHL1* significantly enhanced both the relative expression levels of these genes and L-AsA content in the pulp tissues of ‘Jinyan’ and ‘Ganlv 1’ kiwifruit, compared to the empty vector control (OE-EV). In ‘Jinyan’, gene expression increased by 30.64-, 4.95-, 7.52-, and 19.97-fold, respectively, accompanied by corresponding rises in L-AsA content of 138.60, 62.60, 46.73, and 59.52%. In ‘Ganlv 1’, expression was elevated by 15.02-, 7.06-, 7.62-, and 13.69-fold (Fig. 3A–E), with L-AsA content increasing by 26.34, 9.63, 6.85, and 22.87%, respectively. Conversely, virus-induced gene silencing (VIGS)-mediated silencing markedly suppressed the expression of these genes and reduced L-AsA accumulation relative to the VIGS empty vector control (VIGS-EV). In ‘Jinyan’, relative expression levels declined by 90.87, 20.16, 80.05, and 83.57%, respectively, while L-AsA content decreased by 34.66, 12.73, 17.94, and 38.28%. Similarly, in ‘Ganlv 1’, expression was reduced by 88.31, 13.33, 17.55, and 75.31%, and L-AsA content declined by 30.31, 5.61, 14.19, and 19.37% (Fig. 3F–J). Transient expression assays in kiwifruit provided preliminary evidence that all four candidate genes are involved in the regulation of L-AsA metabolism. Notably, transient modulation of *AebHLH89* expression exerted a more pronounced effect on L-AsA accumulation compared to that of *AePPR*, *AePP2Ab*, or *AePHL1*.

**Figure 4:**
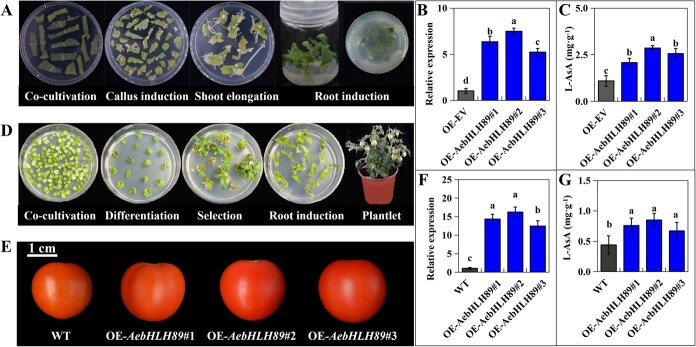
Genetic transformation in tomatoes and kiwifruit. (A) Transgenic lines with overexpression of *AebHLH89* gene in *A. chinensis*; (B and C) relative expression and L-AsA content of *A. chinensis* transgenic line after overexpression of *AebHLH89* gene, respectively; (D and E) three transgenic lines with overexpression of *AebHLH89* gene in ‘Micro Tom’ tomato; (F and G) relative expression and L-AsA content of ‘Micro Tom’ tomato transgenic line after overexpression of *AebHLH89* gene, respectively.

A total of seven transgenic *A. chinensis* lines overexpressing *AebHLH89* were obtained. Among these, three lines with varying expression levels were selected for further analysis. Compared with the OE-EV, these transgenic lines exhibited significantly higher *AebHLH89* expression levels (5.03- to 7.21-fold increase) and elevated L-AsA content (ranging from 60.00 to 89.09%) (Fig. 4A–D). Similarly, following the transformation of ‘Micro Tom’ tomato plants with the *AebHLH89* overexpression construct, 10 positive transgenic lines were acquired. Three lines showing differential expression levels were chosen for subsequent evaluation. Relative to wild-type (WT) plants, these transgenic tomato lines demonstrated markedly increased *AebHLH89* expression (11.99- to 15.62-fold higher) and enhanced L-AsA content (ranging from 52.27 to 93.18%) (Fig. 4E–G).

### Analysis expression of L-ascorbic acid-related gene in the *AebHLH89* regulatory expression line

To investigate whether *AebHLH89* interacts with the promoters of genes involved in the L-AsA metabolic pathway and participates in regulating their expression, we selected 12 genes from this pathway for relative expression analysis using both transiently and stably transformed ‘Micro Tom’ tomato fruit samples (Fig. S6). Reverse transcription-quantitative polymerase chain reaction (RT-qPCR) results demonstrated that overexpression of *AebHLH89* enhanced the expression of genes in the biosynthetic pathway as well as *AeMDHAR* and *AeDHAR* in the recycling pathway, with particularly pronounced effects on *AeGMP1*, *AeGPP1*, *AeGGP3*, and *AeMDHAR*. Specifically, the expression levels of *AeGMP1*, *AeGGP3*, *AeGPP1*, and *AeMDHAR* increased by 372.55–376.79%, 148.91–338.56%, 64.54–222.09%, and 77.69–202.95%, respectively. Conversely, silencing of *AebHLH89* led to downregulation of all examined biosynthetic genes, with expression levels of *AeGMP1*, *AeGPP1*, *AeGGP3*, and *AeMDHAR* reduced by 77.83–84.24%, 13.10–46.52%, 20.15–28.02%, and 36.41–61.87%, respectively. These findings collectively suggest that *AebHLH89* may directly regulate the expression of these four genes.

### AebHLH89 activates the *AeGMP1* promoter and induces interaction.

**Figure 5:**
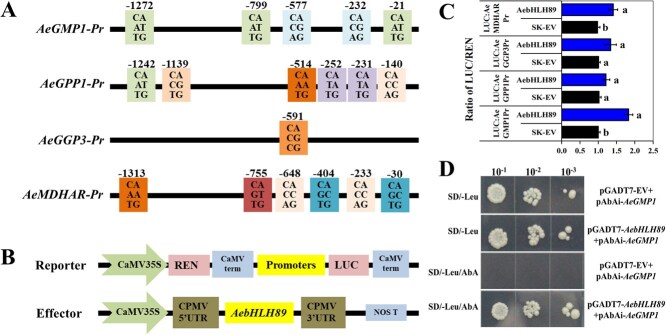
Analysis of AebHLH89 activates the *AeGMP1* promoter and induces interaction. (A) Distribution of predicted cis-acting elements in the promoter fragments of *AeGMP1*, *AeGPP1*, *AeGGP3*, and *AeMDHAR*; (B) vector construction of reporter genes and effectors in the dual-LUC system; (C) relative ratios of LUC/REN in promoter-to-reporter vector combinations in tobacco leaves, with the relative ratios of LUC/REN of each promoter to the empty-loaded combinations used as controls. Different letters indicate the significance (*P* < 0.05) of the difference in the relative ratio of LUC/REN in SK-EV versus SK-AebHLH89 for the same promoter; and (D) Y1H analysis the interactions between AebHLH89 with *AeGMP1* promoter.

Cis-acting element prediction analysis revealed multiple bHLH transcription factor-related cis-acting elements within the 1500 bp promoter region, including binding motifs, such as CAATTG, CACGAG, CACCAG, CATATG, CAGCTG, CACGCG, CACGTG, and CACCAG (Fig. 5A). Coexpression of AebHLH89 significantly enhanced the luciferase (LUC) activity driven by the *AeGMP1* and *AeMDHAR* promoters compared to the empty vector control. In contrast, no significant differences in LUC activity were observed when AebHLH89 was cotransfected with the *AeGGP3* or *AeGPP1* promoters (Fig. 5C). Notably, AebHLH89 activated the *AeGMP1* promoter more strongly (1.83-fold increase) than the *AeMDHAR* promoter (1.41-fold increase), suggesting a preferential regulatory effect on *AeGMP1*. To further assess the potential interaction between AebHLH89 and these promoters, yeast one-hybrid assays (Y1H) were performed. While all yeast strains grew normally on SD/-Leu medium, only the strain harboring the *AeGMP1* promoter-driven reporter (pAbAi-AeGMP1) maintained growth on medium supplemented with 200 ng·ml^−1^ AbA (Fig. 5B and D). This result preliminarily suggests that AebHLH89 bind to the *AeGMP1* promoter *in vivo*.

## Discussion

In this study, leveraging a diverse collection of 216 *A. eriantha* accessions sourced from natural populations with same or similar habitat, we employed GWAS analyses to pinpoint the genetic mutation loci associated with fruit L-AsA traits. Our comprehensive analysis *A. eriantha* population uncovered significant phenotypic variation and extensive genetic diversity in fruit L-AsA traits within the tested population, underscoring the substantial potential for targeted selection and genetic improvement in breeding initiatives.

The high-density and high-quality SNP and InDel markers developed in this study based on whole-genome resequencing are a powerful tool for mining excellent alleles, and also greatly enrich the research of SNPs and InDels in *A. eriantha*. Due to the conserved nature of Bonferroni significance, this study chose a slightly lower GWAS signaling threshold and successfully mined SNP and InDel marker signals and highly associated candidate genes that were significantly associated with the target traits, an approach that has been applied in previous studies [25, 28, 29, 36]. In general, natural population resources are often used to perform GWAS because natural populations undergo multiple generations of recombination and they possess smaller interlocking blocks, which allows for more precise localization of functional genes [18]. It is noteworthy that the number of identified SNPs (1 790 395) in this study is substantially lower than that of InDels (7 400 509), a pattern that deviates from the typically observed predominance of SNPs over InDels in plant genomes, as exemplified by a recent *A. eriantha* study reporting approximately 11.3 million SNPs and 1.4 million InDels [37]. This discrepancy likely stems from a combination of factors inherent to the variant calling pipeline and the genetic characteristics of the germplasm panel. First, the variant calling stringency, particularly the quality filters applied to low-confidence variants, may have disproportionately filtered out SNPs due to sequencing errors or alignment ambiguities in repetitive regions, while InDels, especially those validated by read pairings, may have been retained more conservatively. Second, the use of different reference genome assemblies and annotation versions can significantly impact alignment efficiency and, consequently, variant discovery rates. Finally, the *A. eriantha* germplasm accessions used in this study may harbor a higher intrinsic frequency of InDels relative to SNPs, potentially reflecting unique genomic structural variations or recent evolutionary dynamics within this population. In this study, a high-density set of high-quality SNP and InDel markers provided a robust foundation for GWAS.

Additionally, the tested materials exhibited substantial phenotypic variability in fruit quality traits, making them well-suited for GWAS. To minimize false positives and enhance analytical accuracy, we systematically evaluated the associations of L-AsA content across four models, ultimately selecting the MLM-QK model—which accounts for both population structure and relatedness—for downstream analysis. The Q–Q plots based on the MLM-QK model showed close agreement between observed and expected values, confirming effective correction for population stratification *via* PCA. Furthermore, since intrinsic fruit quality traits are predominantly governed by genetic factors, the candidate genes identified here differed from those previously reported. Notably, the four candidate genes further identified through functional analysis of SNPs and InDels significantly associated with L-AsA *via* GWAS screening share functional connections with known regulators of fruit quality. Their regulatory roles were further validated through relative expression and functional analyses, reinforcing the reliability of our GWAS results.

Notably, we identified an SNP cluster (comprising four SNPs) on chromosome 23 that was significantly associated with L-AsA levels. This cluster, which included one highly significant SNP (−log_10_(*P*) > 7.54) and three additional significant SNPs (−log_10_(*P*) > 6.27), indicated the presence of regulatory loci for L-AsA contents in *A. eriantha* fruits. Prior studies have mapped genes involved in L-AsA metabolism to chromosome 26 in the hybrid offspring of *A. hairy* × *A. delicious* × *A. sinensis* [38] and to chromosome 29 in the natural hybrid offspring of *A. eriantha* cv. ‘White’ [18]. Three genes [*AePPR* (*Chr23g461*), *AePP2Ab* (*Chr23g463*), and *AePHL1* (*Chr23g467*)] potentially associated with L-AsA metabolism were jointly identified within a 50 kb upstream and downstream region of the significant SNPs and InDels. Concurrently, within the candidate interval at SNP locus Chr17:2255503, *AebHLH89* was found to potentially participate in L-AsA metabolic regulation.

In maize, *ZmbHLH55* coexpresses with L-galactose pathway genes *ZmPGI2*, *ZmGME1*, and *ZmGLDH*. *ZmbHLH55* knockdown mutants show reduced L-AsA under salt stress, whereas *Arabidopsis* overexpressing *ZmbHLH55* has higher L-AsA levels under the same conditions [32]. Interestingly, the transient transformation of *AebHLH89* overexpressed and silenced expression in kiwifruit fruits and *AebHLH89* overexpression stable transfection in tomatoes, and *A. chinensis* in this study yielded similar results, validating *AebHLH89* as a positive regulator of L-AsA accumulation. Simultaneously, Y1H interaction validation, transcriptional activation functional analysis in plant cells, and cocorrelation of expression patterns provide multifaceted evidence supporting the conclusion that AebHLH89 regulates *AeGMP1* transcription by binding to its promoter, thereby inducing the biosynthetic pathway to promote L-AsA synthesis and accumulation. GMP encodes GDP-mannose pyrophosphorylase, a key enzyme catalyzing the interconversion of D-mannose-1-phosphate and GDP-D-mannose in the L-galactose pathway of L-AsA biosynthesis. L-AsA levels in *Arabidopsis GMP* mutants (*VTC1*) were significantly lower than in WT plants, showing a 75% reduction [39]. Silencing and overexpression of tomato *GMP3* resulted in decreased and increased L-AsA levels, respectively [40]. Overexpression of *GmGMP1* significantly elevated L-AsA content in both homologous transformation in soybean and heterologous transformation in *Arabidopsis* [41]. These studies collectively confirm GMP’s crucial role in L-AsA biosynthesis. In this study, the *AeGMP1* overexpression fusion vector was stably homoeotransformed into *A. chinensis* callus, significantly increasing L-AsA content in leaves of positive transformants (These data have not yet been publicly released.) This result further solidifies GMP’s essential function in L-AsA biosynthesis.

*PP2Ab* serves as the catalytic regulatory subunit B of PP2A. It regulates plant cell development and stress responses [33]. Its substrates include protein kinases and transcription factors, which it dephosphorylates to release inorganic phosphate (Pi), thereby modulating signaling pathways. Like PTP, PP2A directly dephosphorylates receptor protein kinases, thereby regulating ABA signaling. The PP6 holoenzyme (containing FyPP1 and FyPP3) directly dephosphorylates ABSCISIC ACID INSENSITIVE5 (ABI5), modulating ABA signaling [21, 42]. PTPN hydrolyzes GDP/GMP/dGMP/IMP/dIMP to release Pi, mediating crosstalk between ABA signaling and L-AsA biosynthesis and enhancing drought resistance [7]. *VTC2* and *VTC5* require Pi to convert GDP-L-galactose to L-galactose-1-phosphate, a rate-limiting step in L-AsA synthesis. This step promotes L-AsA accumulation [7]. The PP2A subunit composition critically influences ROS signaling. The *Arabidopsis PP2Ab’γζ* double mutant exhibits enhanced tolerance to prolonged abiotic stress, including high light, high temperature, and drought [43]. Ascorbate peroxidase 2 (*APX2*) responds to signals from the photosynthetic electron transport chain, ABA, and metabolic pathways. In the *PP2Ab’γζ* double mutant, *APX2* transcript levels increased under strong light, enhancing oxidative stress tolerance [43]. APX is critical for plant growth, development, and stress responses. As a key enzyme in the AsA-GSH cycle, it regulates L-AsA homeostasis [31]. In the *PP2Ab’γζ* double mutant, elevated APX2 expression disrupted L-AsA homeostasis in the AsA-GSH cycle, triggering L-AsA accumulation to mitigate photooxidative stress damage. In *Arabidopsis*, PP2Ab’γ mediates photooxidative stress responses *via* ROS signaling and metabolic homeostasis regulation [43].

PPR proteins, as RNA-binding trans-acting factors, regulate plant stress responses by modulating organelle RNA metabolism. In rice, many *PPR* genes are upregulated under abiotic stress, enhancing stress resistance and tolerance [33]. The cytoplasmic-nuclear PPR protein SOAR1 negatively regulates ABA signaling during seed germination and early growth but acts as a positive regulator of abiotic stress responses [44]. The *Arabidopsis* PPR protein FOR GERMINATION ON NaCl (PGN) also plays a role in plant defense and abiotic stress responses [44]. PGN inactivation causes sensitivity to ABA, glucose, and salt, with increased reactive oxygen species in seedlings under salt stress. Inhibiting ABA synthesis or signaling partially alleviates PGN’s glucose sensitivity [44]. PPR proteins mitigate stress damage by modulating ABA signaling. ABA is a key regulator of stress responses and often acts as a negative regulator of plant defense mechanisms. Under stress, ABA accumulation often triggers L-AsA increases *via* glutathione to maintain cellular homeostasis [7].

Additionally, *Chr23g467* encodes PHL1, a PHR1-family transcription factor with MYB and helix domains, which regulates plant Pi metabolism [35]. *PHR* transcription factors integrate environmental phosphate signals, optimize Pi use efficiency by regulating root phosphate transporter genes, and help plants adapt to low-Pi conditions [45]. As noted, *VTC2* and *VTC5* in the L-galactose pathway depend on Pi to convert GDP-L-galactose to L-galactose-1-phosphate [7]. *PHL1* positively regulates L-AsA accumulation by modulating endogenous Pi levels in plant Pi metabolism. Based on these findings, we hypothesize that these genes significantly contribute to L-AsA regulation. Our results offer novel insights into strategies for maintaining high L-AsA levels in kiwifruit.

## Conclusions

L-AsA functions as an essential antioxidant, cofactor, and redox buffer in both plants and humans, playing a central role in cellular redox balance, enzymatic regulation, and stress resistance. In this study, GWAS analysis of 216 *A. eriantha* accessions identified SNPs and InDels significantly associated with L-AsA content and revealed candidate genes (*AebHLH89*, *AePPR*, *AePP2Ab*, and *AePHL1*) involved in its metabolism. Among these, the transcription factor *AebHLH89* was characterized as a positive regulator of L-AsA biosynthesis. Transient expression in kiwifruit fruits and stable transformation in both *A. chinensis* and tomato significantly enhanced L-AsA levels. Mechanistically, AebHLH89 could binds to the promoter of *AeGMP1* and activates its transcription, thereby promoting the L-AsA biosynthetic pathway. These findings advance the understanding of L-AsA regulatory mechanisms and provide a basis for molecular breeding in kiwifruit.

## Materials and methods

### Plant materials and determination of fruit L-ascorbic acid content

GWAS was conducted using 216 *A. eriantha* individuals, which were selected from a previously reported natural population with similar growth environments of 236 *A. eriantha* accessions sourced from the Luoxiao Mountains in Ganzhou City, Jiangxi Province, China (114°1′1.2″–114°3′9.2″ E, 25°46′24.6″–25°48′7.2″ N, altitude 1092.98–1272.19 m) in mid-October 2022 (Table S6) [29]. Here, 12–30 healthy and undamaged physiologically mature fruits (with a soluble solid content of approximately 6.5%) and healthy and mature leaf replicates were harvested centrally from each wild *A. eriantha* individual plant; the distance between individual plants was at least 50 m [46]. A total of 216 individual plants of *A. eriantha* fruits and leaves were intensively harvested from wild populations. *A. eriantha* fruits of the 216 individuals were stored at room temperature (25 ± 2°C), and allowed to ripen until they reached an edible state (10 N fruit firmness). These ripe fruits were subsequently used to measure L-AsA content. High-performance liquid chromatography (HPLC, Shimadzu LC-10A) was employed to quantify L-AsA content according to previously described [30].

### DNA extraction, whole-genome resequencing, and variant calling

Genomic DNA was extracted and evaluated using previously described methods [46]. The DNA was randomly fragmented into 300–500 bp fragments using a Bioruptor. The sequencing library was constructed using the VAHTS Universal DNA Library Prep Kit for MGI (NDM607, Vazyme, Nanjing, China), following the manufacturer’s recommendations. Sequencing was performed on the Illumina NovaSeq 6000 platform (Illumina, California, USA) with a PE150 strategy. Whole-genome resequencing was conducted by Wuhan IGENEBOOK Biotechnology Co., Ltd. (http://www.igenebook.com). The genome of *A. eriantha* cv. ‘Ganlv 1’ was used as a reference genome [31]. Raw sequencing data were assessed for quality using FastQC (https://github.com/s-andrews/FastQC) [47]. Clean data were obtained through quality control and trimming with Fastp (https://github.com/OpenGene/fastp) and were subsequently mapped to the reference genome. The resulting SAM files were converted to BAM format using Samtools [48]. Duplicates were marked, and SNP/InDel calling were performed using GATK [49]. High-quality SNPs and InDels were filtered based on average filtering criteria and were used for downstream GWAS analyses.

### Population structure and linkage disequilibrium analysis

Admixture v1.3 was used to determine the population structure. We employed 2–5 of clusters (*K* value) for clustering. The *K* value with the minimum cross-validation error was taken as the optimal number of clusters [27]. The phylogenetic tree was constructed using MEGA-X. The bootstrap values were obtained after 500 calculations, and the phylogenetic tree of 216 *A. eriantha* accessions was obtained. To further understand the classification of the population under different subgroups and the lineage composition of each sample, Pophelper v2.2.7 was used to create a histogram of the genetic composition of each sample in each subgroup [50]. PCA was performed using GCTA v1.93.2 [50] based on the filtered SNPs. LD analysis was performed based on high-quality SNPs obtained after filtering. Plink v2.0 and PopldDecay v3.41 were used to calculate the *r*^2^ between SNP markers [51].

### Genome-wide association studies

GWAS was conducted to identify associations between L-AsA traits and SNPs using GEMMA v0.98.1 (University of Chicago, Chicago, USA). An association value (*P*) was calculated for each SNP and InDel, and the significant *P*-values were corrected using Benjamini–Hochberg method was used to correct for the significance of the *P*-value and two thresholds were set –log_10_(*P*) > 6.27 (0.05 level of significance) and –log_10_(*P*) > 7.54 (0.01 level of significance) [36, 52]. The corrected *P*-values were visualized using Manhattan and Q–Q plots after applying –log_10_ transformation. For GWAS analyses, both general linear models (GLM and GLM-Q) and mixed linear models (MLM-K and MLM-QK) were employed, with results interpreted based on the Manhattan and Q–Q plots. SNPs with significant correlation (−log_10_(*P*) > 6.27) were identified as candidate loci for specific traits, and genes localized within 50 kb upstream and downstream of these loci were identified as candidate genes and genetically annotated through the KGD database (http://kiwifruitgenome.org/) and NCBI (https://www.ncbi.nlm.nih.gov/) [46].

### Candidate gene mining and reverse transcription-quantitative polymerase chain reaction validation

Candidate genes located within significant association SNP/InDel loci (−log_10_(*P*) > 6.27) and within a 50 kb region upstream and downstream of these loci [46] were selected for expression analysis *via* RT-qPCR. These analyses were performed during fruit development in ‘Ganmi 6’, a commercially cultivated kiwifruit variety independently bred by kiwifruit institute of Jiangxi Agricultural University [53]. RNA extraction, reverse transcription into cDNA, and RT-qPCR were conducted as previously described [54]. RT-qPCR primers are listed in Table S7. The analysis included three biological replicates and three technical replicates. Relative expression levels of each gene were calculated using the 2^−ΔΔct^ method, with *Actin* and *GAPDH* serving as the reference genes.

### Gene clone and expression vector construction

The full-length coding sequence of *AebHLH89*, *AePPR*, *AePP2Ab*, *AePHL1*, and *AeGMP1* without stop codon was cloned from cDNA template of wild *A. eriantha* CSA19 and inserted into the overexpression vector pCAMBIA1300-35S-EGFP by homologous recombination method using the Trelief SoSoo Clonging Kit (Beijing Tsingke Xinye Biotechnology Co. Ltd., Beijing, China) to form integration expression module. Using the same method, the expression modules pCAMBIA1300-35S-EGFP-*AeGMP1*, pGreenII62-SK-*AebHLH89*, and pGADT7-*AebHLH89* were constructed. Using genomic DNA from wild *A. eriantha* CSA19 as a template, the 1500 bp downstream target gene promoter sequence was cloned. The pGreenII0800-LUC-*AeGMP1*, *AeGPP1*, *AeGGP3*, *AeMDHAR*, and pAbAi-*AeGMP1* expression modules were constructed *via* homologous recombination. The VIGS tool of Sol Genomics was used to select a specific 300 bp partial sequences of *AebHLH89*, *AePPR*, *AePP2Ab*, and *AePHL1*, and the 300 bp silencing fragment was inserted into the silencing expression vector pTRV2 to form pTRV2-*AebHLH89*/*AePPR*/*AePP2Ab*/*AePHL1* integration expression module [55, 56].

### Kiwifruit transient transformation

The plasmids of overexpression integration expression module and silencing integration expression module were transformed into *Agrobacterium tumefaciens* GV3101. The transformed *Agrobacterium*s were then used for transient transformation of healthy fruits from ‘Jinyan’ (*A. eriantha* × *A. chinensis*) at 120 DAF and ‘Ganlv 1’ at 105 DAF, following a previously reported *Agrobacterium*-mediated method [18]. The experiment was performed with three biological replicates per group, each containing 20 injected fruits. After injection, the fruits were stored in darkness at 25°C for 6 days. Then samples of the injection site were collected, frozen in liquid nitrogen and stored at −80°C for the detection of L-AsA content and the relative expression levels of genes associated with its metabolism. All primers used for plasmid construction are listed in Table S7.

### Genetic transformation in kiwifruit and tomato

Simultaneously, *A. tumefaciens* GV3101 containing the pCAMBIA1300-35S-EGFP-*AebHLH89* expression module was used for transient transformation and subsequent infection of ‘Micro Tom’ tomato and sterile leaves of *A. chinensis*. Genetic transformation of tomato and kiwifruit was performed according to previously described methods. Genomic DNA was extracted using the aforementioned method [46], and positive lines were identified *via* PCR. Primers used for PCR positive identification are listed in Table S5. Harvest red-ripe fruits from positive tomato lines for relative gene expression analysis and L-AsA content detection, and collect red-ripe transgenic and WT tomato seeds.

### Subcellular localization

Transform pCAMBIA1300-35S-EGFP-*AebHLH89* expressing green fluorescent protein (GFP) and the control vector (pCAMBIA1300-35S-EGFP) into *A. tumefaciens* GV3101, then inject the solution onto the abaxial surface of approximately 4-week-old tobacco leaves. After incubating for 2–3 days in a growth chamber, GFP signals in the leaves were observed using a full-spectrum laser confocal microscope (Olympus Corporation, Tokyo, Japan).

### Dual-luciferase assay

The cis-acting elements in the promoter sequences of the target genes were predicted using the PlantCARE website (http://bioinformatics.psb.ugent.be/webtools/plantcare/html/) and the JASPAR database (https://jaspar.elixir.no/). Simultaneously, the previously described pGreenII62-SK-*AebHLH89* vector was employed as an effector construct, while pGreenII0800-LUC vectors carrying *AeGMP1*, *AeGPP1*, *AeGGP3*, or *AeMDHAR* were used as reporter constructs. The reporter and effector constructs were cointroduced into *A. tumefaciens* GV3101, which was then infiltrated into leaves of 4-week-old tobacco plants following a previously described method. Firefly LUC and Renilla LUC (REN) activities were measured for dual-LUC assay.

### Yeast one-hybrid assay

The previously described pGADT7-*AebHLH89* vector was employed as a prey vector, while pGreenII0800-LUC vectors carrying *AeGMP1* was used as bait vector. The bait plasmid pAbAi-pro*AeGMP1* was transformed into the Y1H Gold yeast strain using the Super Yeast Competent Cell Preparation and Transformation Kit (Coolaber, Beijing, China). The transformed yeast cells were plated on SD/-Ura medium and incubated at 28°C for 3–5 days. Single colonies were then selected and verified by colony PCR using the Yeast Genome Colony PCR Kit (Coolaber, Beijing, China). Positive clones were cultured, and the OD600 was adjusted to 0.002 with 0.9% NaCl. Aliquots of 100 μl were spread on SD/−Ura agar plates containing 0, 50, 100, 200, or 300 ng·ml^−1^ of Aureobasidin A (AbA) to assess bait autoactivation and determine the minimum AbA concentration required to suppress it. For the Y1H, the prey plasmids pGADT7-*AebHLH89* and the empty pGADT7 vector were separately transformed into yeast harboring pAbAi-*AeGMP1* using a yeast transformation kit. The transformants were plated on SD/−Leu medium supplemented with the minimal autoactivation-inhibiting concentration of AbA, with SD/-Leu plates without AbA serving as the control. Protein–DNA interaction between AebHLH89 and *AeGMP1* was evaluated based on colony growth under selective conditions.

### Statistical analysis

Data were collated and analyzed using Microsoft Excel 2019 to calculate maximum, minimum, mean, SD, CV, and Shannon–Wiener information index (*H*′). Box plots and scatter plots analyses were performed using OriginPro 2024b (OriginLab Inc., Northampton, MA, USA).

## Supplementary Material

Web_Material_uhag116

## Data Availability

The whole-genome sequencing raw data have been uploaded to national genomics data center (NGDC, https://ngdc.cncb.ac.cn/) with the accession number CRA015678.
