# Supplementary material for: The transcription factor AebHLH89 activates AeGMP1 transcription to regulate L-ascorbic acid accumulation in kiwifruit (Actinidia eriantha) revealed by genome-wide association study
Source: Hortic Res. 2026 Apr 6;13(7):uhag116. doi: 10.1093/hr/uhag116 (PMC13341127; doi:10.1093/hr/uhag116)
Supplement: Web_Material_uhag116 [file web_material_uhag116.zip › 2.9-Supplementary Figures-for R2.docx]

**Supplementary Figures:**


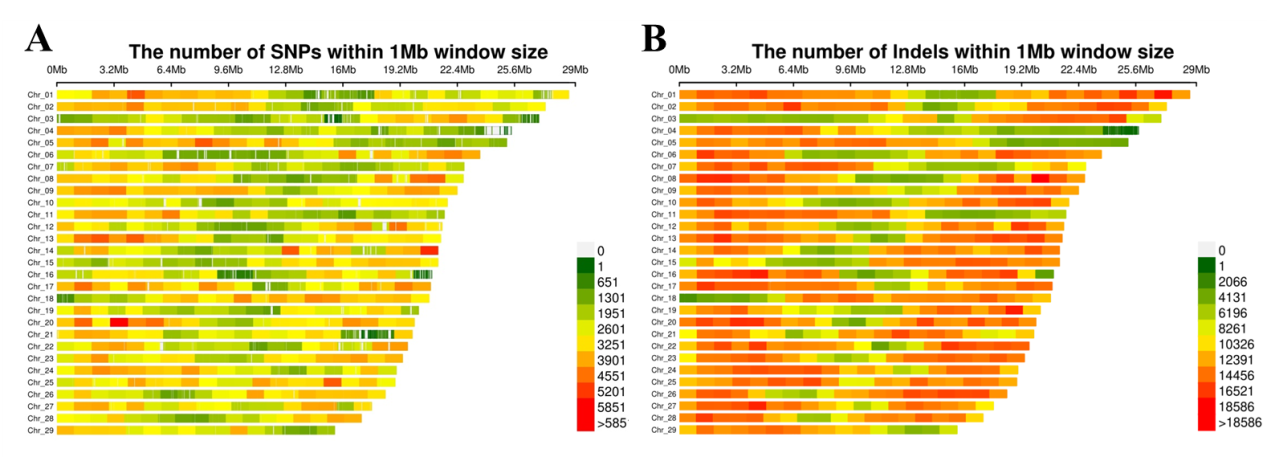


**Figure S1. Distribution of SNPs (A) and InDels on each chromosome (B).**

**
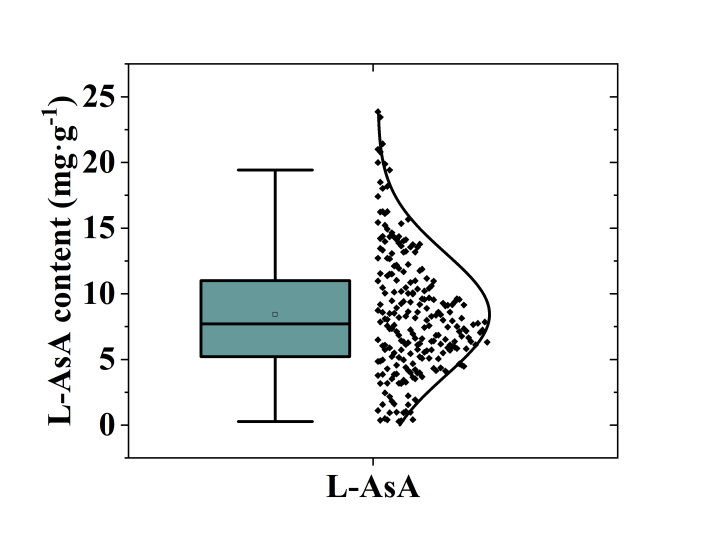
**

**Figure S2. Box plots and scatter plots of L-AsA traits.**

**
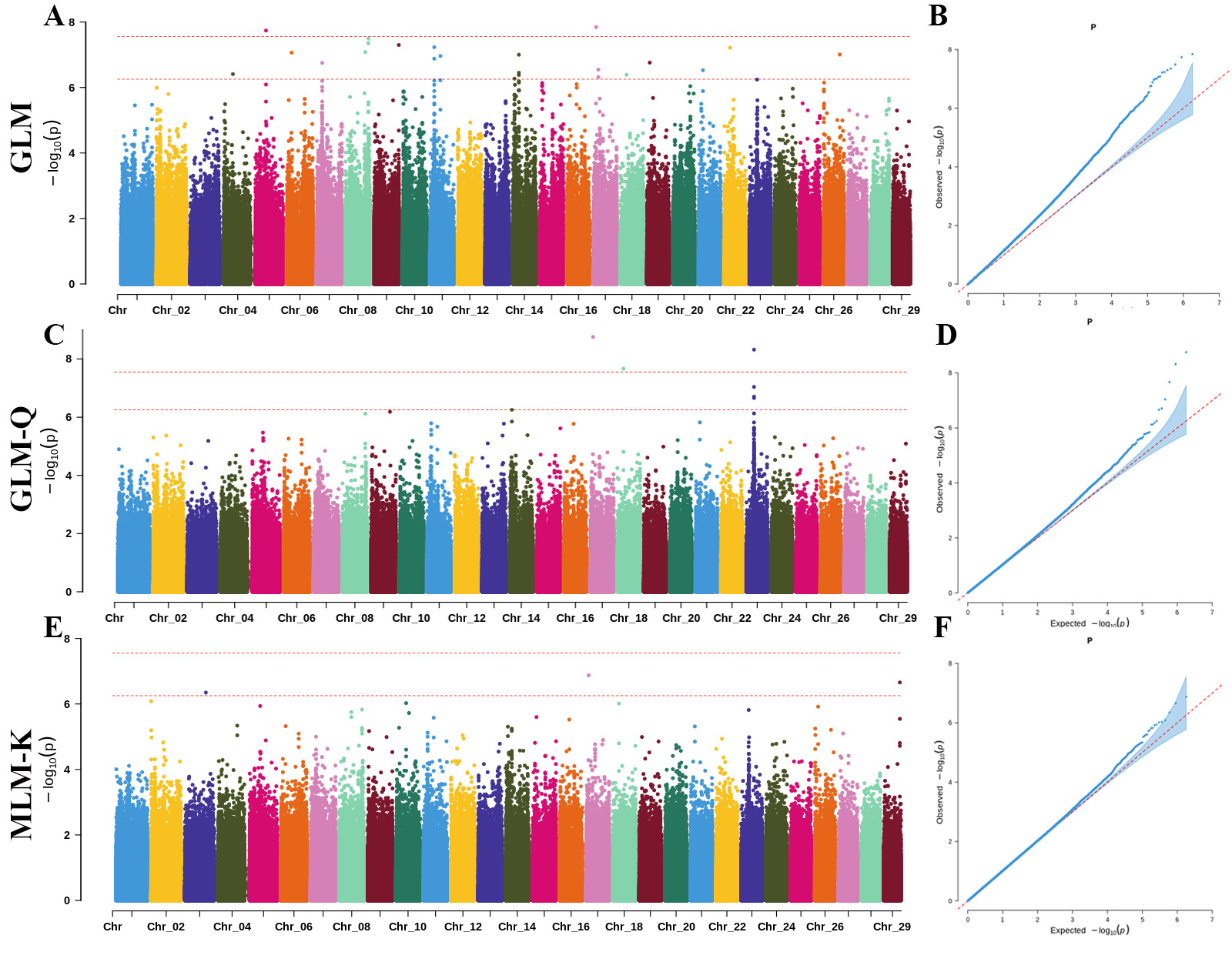
**

**Figure S3. GWAS analyses of L-AsA trait in 216 *A. eriantha* accessions based on based on SNP markers.** (A, B) Manhattan plots (A) and Q-Q plots (B) for L-AsA-associated SNPs based on GLM. (C, D) Manhattan plots (C) and Q-Q plots (D) based on GLM-Q. (E, F) Manhattan plots (E) and Q-Q plots (F) based on MLM-K.

**
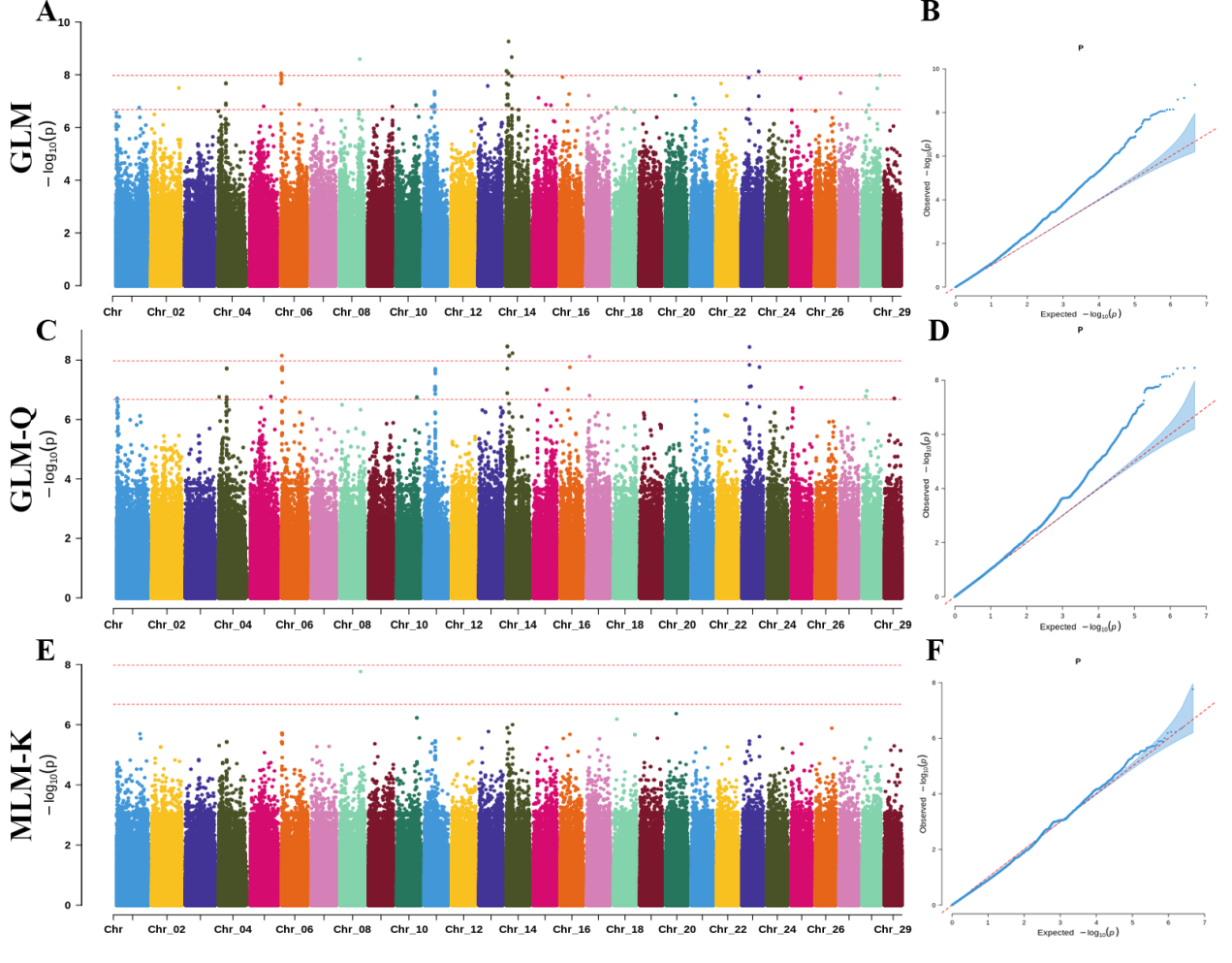
**

**Figure S4. GWAS analyses of L-AsA trait in 216 *A. eriantha* accessions based on based on InDel markers.** (A, B) Manhattan plots (A) and Q-Q plots (B) for L-AsA-associated InDels based on GLM. (C, D) Manhattan plots (C) and Q-Q plots (D) based on GLM-Q. (E, F) Manhattan plots (E) and Q-Q plots (F) based on MLM-K.


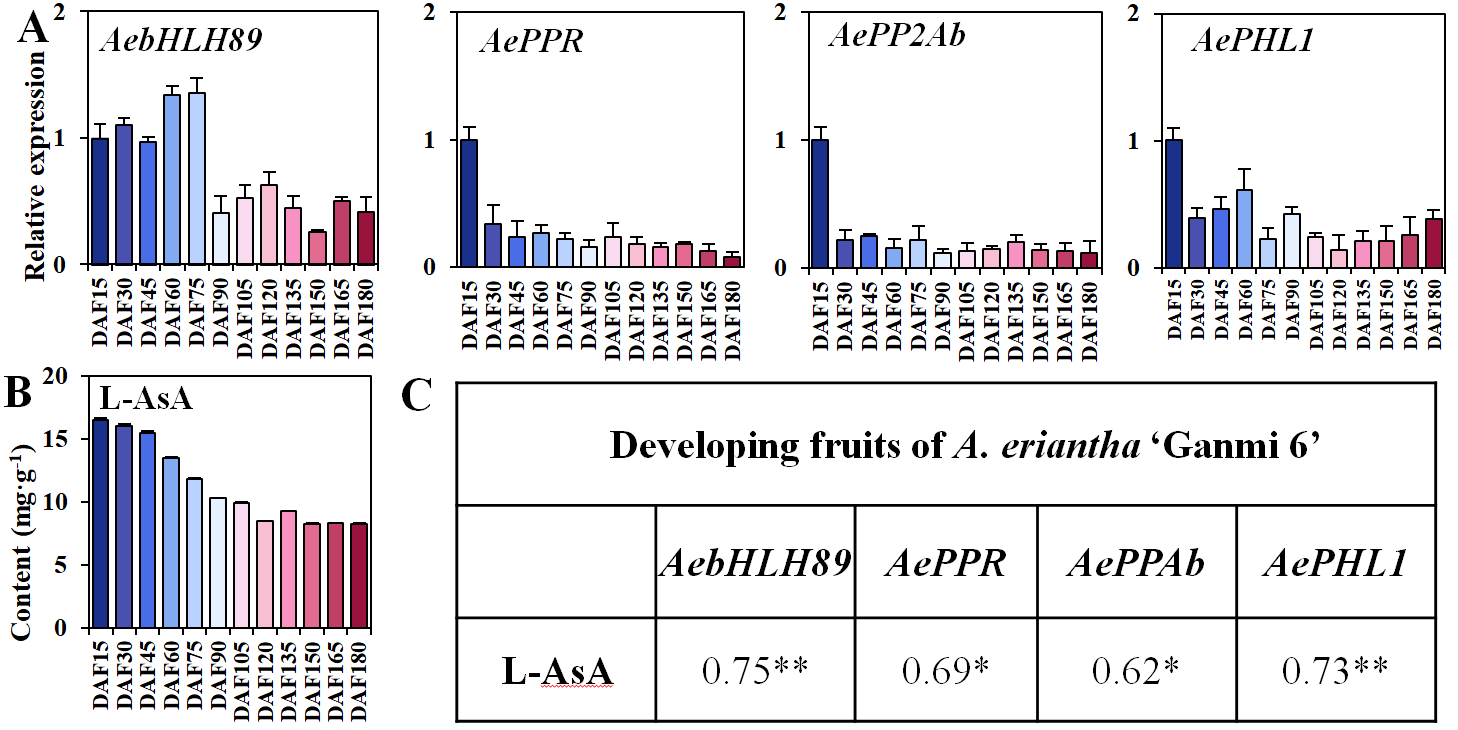


**Figure S5. Relative expression levels of four candidate genes and L-AsA contents in fruits of ‘Ganmi 6’ at different developmental stages (A, B), with correlation analysis shown in (C).** * and ** represent statistical significance at *P*<0.05 and *P*<0.01, respectively.

**
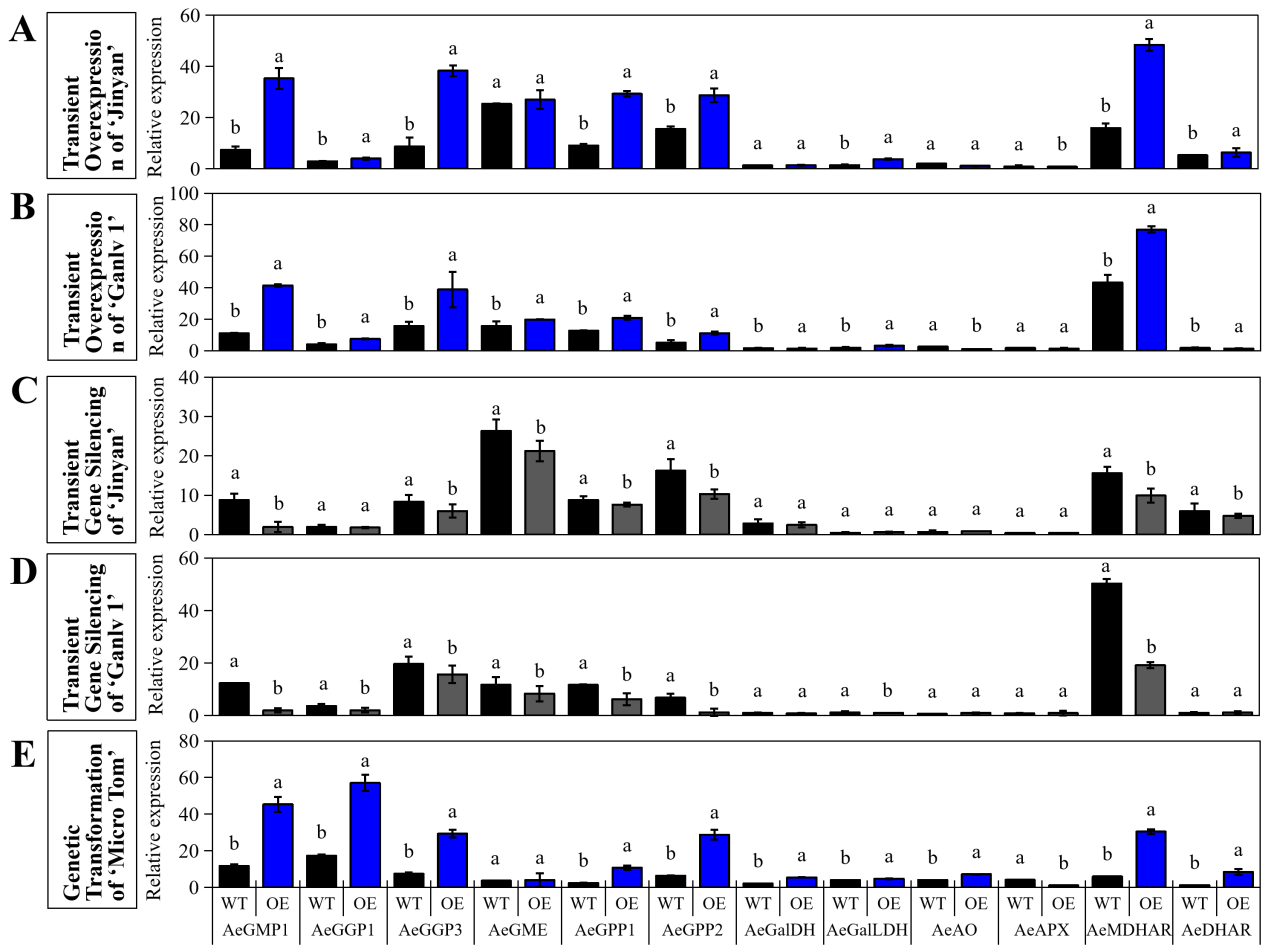
**

**Figure S6. Expression of L-AsA metabolism pathway related genes regulation by transient transformation and stable transformation of *AebHLH89* gene.** (A) transient overexpression in ‘Jinyan’ fruit; (B) transient overexpression in ‘Ganlv 1’ fruit; (C) transient silent expression in ‘Jinyan’ fruit; (D) transient silent overexpression in ‘Ganlv 1’ fruit; (E) stable overexpression in ‘Micro Tom’ tomato; different letters indicated the significant differences (*P*<0.05) between WT and overexpression strains for the same gene. Black bars represent empty loads, blue bars represent over-expressed coefficients, and the gray columns represent silent systems.
